# CD8^+^ T cell infiltration and proliferation in the brainstem during experimental cerebral malaria

**DOI:** 10.1111/cns.14431

**Published:** 2023-09-12

**Authors:** Jun Wang, Qinghao Zhu, Yan Shen, Jiao Liang, Yi Wang, Yuxiao Huang, Guodong Tong, Xu Wang, Ningning Zhang, Kangjie Yu, Yinghui Li, Ya Zhao

**Affiliations:** ^1^ Department of Medical Microbiology and Parasitology Fourth Military Medical University Xi'an China; ^2^ College of Life Sciences Northwest University Xi'an China; ^3^ School of Basic Medical Sciences Fourth Military Medical University Xi'an China; ^4^ Department of Pathology Air Force Hospital of Eastern Theater Nanjing China

**Keywords:** astrocytes, brain‐infiltrated CD8^+^ T cell, brainstem, experimental cerebral malaria, PD‐1/PD‐L1 pathway, single‐cell RNA sequencing

## Abstract

**Introduction:**

Cerebral malaria (CM) is a lethal neuroinflammatory disease caused by *Plasmodium* infection. Immune cells and brain parenchyma cells contribute to the pathogenesis of CM. However, a systematic examination of the changes that occur in the brain parenchyma region during CM at the single‐cell resolution is still poorly studied.

**Aims:**

To explore cell composition and CD8^+^ T cell infiltration, single‐cell RNA sequencing (scRNA‐seq) was performed on the brainstems of healthy and experimental cerebral malaria (ECM) mice. Then CD8^+^ T cell infiltration was confirmed by flow cytometry and immunofluorescence assays. Subsequently, the characteristics of the brain‐infiltrated CD8^+^ T cells were analyzed. Finally, the interactions between parenchyma cells and brain‐infiltrated CD8^+^ T cells were studied with an astrocytes‐CD8^+^ T cell cocultured model.

**Results:**

The brainstem is the most severely damaged site during ECM. ScRNA‐seq revealed a large number of CD8^+^ T cells infiltrating into the brainstem in ECM mice. Brain‐infiltrated CD8^+^ T cells were highly activated according to scRNA‐seq, immunofluorescence, and flow cytometry assays. Further analysis found a subset of ki‐67^+^ CD8^+^ T cells that have a higher transcriptional level of genes related to T cell function, activation, and proliferation, suggesting that they were exposed to specific antigens presented by brain parenchyma cells. Brain‐infiltrated CD8^+^ T cells were the only prominent source of IFN‐γ in this single‐cell analysis. Astrocytes, which have a high interferon response, act as cross‐presenting cells to recruit and re‐activate brain‐infiltrated CD8^+^ T cells. We also found that brain‐infiltrated CD8^+^ T cells were highly expressed immune checkpoint molecule PD‐1, while parenchyma cells showed up‐regulation of PD‐L1 after infection.

**Conclusions:**

These findings reveal a novel interaction between brain‐infiltrated CD8^+^ T cells and parenchyma cells in the ECM brainstem, suggesting that the PD‐1/PD‐L1 signal pathway is a promising adjunctive therapeutic strategy for ECM targeting over‐activated CD8^+^ T cells.

## Graphical abstract

Brain‐infiltrated CD8^+^ T cells are highly activated and cytotoxic. Activated astrocytes may act as cross‐presenting cells during ECM. Activated CD8^+^ T cells and PD‐1 signaling are therapeutic targets for CM.

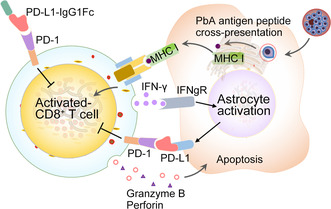

## 1 INTRODUCTION

Cerebral malaria (CM), caused by *Plasmodium falciparum* (*P.f*) infection, is one of the most dangerous complications of malaria infection.^1^, ^2^ Disruption of the blood‐brain barrier (BBB), cerebral edema, and neurological symptoms are hallmarks of CM.^3^, ^4^, ^5^ Although a variety of immune cells are involved in the immunopathological damage during BBB breakdown, CD8^+^ T cell depletion^6^, ^7^ or functional disruption,^8^, ^9^ even just 1 day before the onset of the neurological symptoms, could completely abrogate this disease in a mouse model named experimental cerebral malaria (ECM). Parasite‐specific CD8^+^ T cells mediate the destruction of brain microvascular endothelial cells (BMECs), which is directly responsible for the loss of BBB integrity observed in the ECM.^8^, ^9^, ^10^ Besides BMECs, brain parenchyma cells are also targets of brain‐infiltrated CD8^+^ T cells and play an important role in brain inflammation and central nervous system (CNS) injury.^11^, ^12^ Although the pathogenesis of human CM is not identical to that of ECM, brain‐infiltrated cytotoxic CD8^+^ T cells play a key role in the pathogenesis of human CM, such as BBB breakdown and CNS inflammation.^13^, ^14^

Parenchyma cells may actively modulate the immune response in the CNS, which has been considered an immune‐privileged site.^15^, ^16^, ^17^ Infection‐induced neuroinflammation, within which plays a critical role in defense against infection. However, the chronic presence of activated T cells may be disastrous for this enclosed tissue.^18^, ^19^ Therefore, in addition to the beneficial anti‐infection effects of the pro‐inflammatory response, suppression of neuroinflammation is equally essential for limiting tissue damage and preserving neurological function. An important anti‐inflammatory pathway is the PD‐1/PD‐L1 pathway. Several studies from post‐encephalitic brains suggest that glial cells inhibit CD8^+^ T cell activation through upregulation of the PD‐1/PD‐L1 pathway.^20^, ^21^, ^22^ However, a systematic examination of the changes that occur in the brain parenchyma during CM at single‐cell resolution has not yet been performed.

In the present study, we employed single‐cell RNA sequencing (scRNA‐seq) to characterize the transcriptional profiles of the brainstem from CM and healthy mice and identify potential ways in which brain parenchyma cells and brain‐infiltrated CD8^+^ T cells may communicate and interact with each other. These interactions could illuminate novel therapeutic strategies for the treatment of CM and contribute to our understanding of this complex disease.

## 2 RESULTS

### 2.1 The brainstem is the most severely damaged site in the pathogenesis of ECM

**FIGURE 1:**
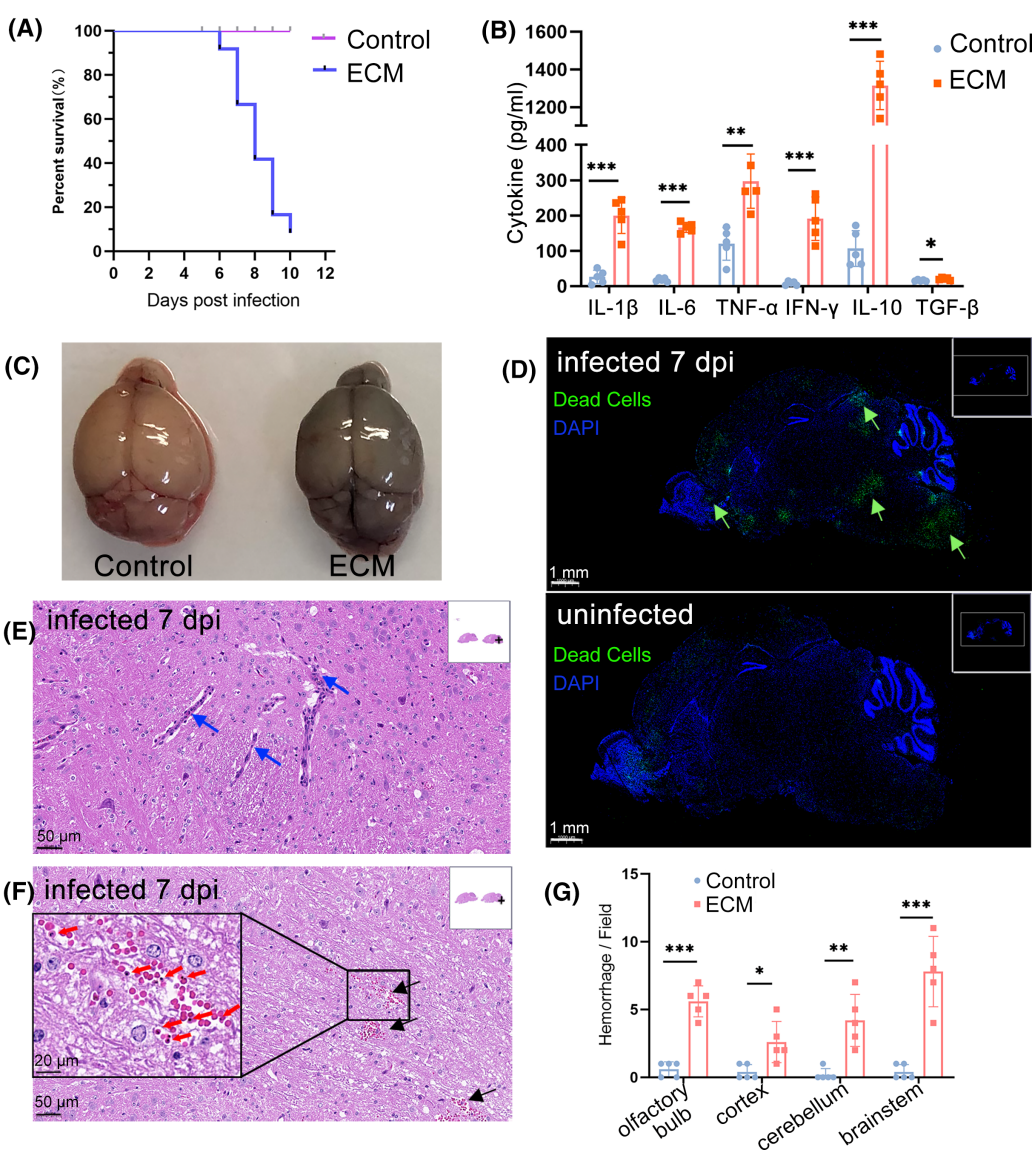
Experimental cerebral malaria (ECM) is associated with severe brainstem pathology. (A) Survival curves of C57BL/6 mice infected with PbA, *n* = 11 mice. (B) Serum IL‐1β, IL‐6, TNF‐α, IFN‐γ, IL‐10, and TGF‐β concentrations of ECM mice were detected by ELISA at 7 dpi. Data are mean ± SEM. Each dot represents one replication, *n* = 5. *p < 0.05, **p < 0.01, ***p < 0.001. (C) Evans blue permeability assay with healthy and symptomatic ECM mice brain at 7 dpi. (D) TUNEL staining for apoptotic cells in ECM mice and healthy mice brain sections; the nucleus was stained with blue and dead cells were stained with green. Scale bar, 1 mm. The green arrows showed apoptotic cells in ECM brain sections. Hematoxylin and eosin (H&E) staining of brain sections of ECM mice (E, F) to detect lymphocyte infiltration (E) and pRBCs' sequestration or hemorrhages (F). Scale bar, 50 μm. Blue arrows indicate lymphocyte infiltration, black arrows indicate pRBCs sequestration, and red arrows indicate brain‐infiltrated pRBCs. (G) Quantitative results of hemorrhage in different brain regions of ECM mice. Data are represented as mean ± SEM, *n* = 5 fields/region, *p < 0.05, **p < 0.01, ***p < 0.001.

Experimental cerebral malaria (ECM) is an acute, fatal central nervous system (CNS) disease (Figure 1A) that is accompanied by severe cytokine release syndrome (CRS) (Figure 1B). An Evans blue (EB) permeability assay with healthy and symptomatic ECM mice at 7 days post‐infection (dpi) showed that the blood‐brain barrier (BBB) breakdown is one of the most remarkable features of ECM (Figure 1C). The CNS has a complex structure and functional partitions, so we performed a TdT‐mediated dUTP‐biotin nick end labeling (TUNEL) assay to reveal apoptotic cells in the ECM brain at 7 dpi, which revealed an inconsistent pathological pattern in the brain. Cell apoptosis was observed in multiple regions (olfactory bulb, cortex, cerebellum, and brainstem) in the ECM brain, but the brainstem and olfactory bulb showed more serious damage compared to other sites (Figure 1D). Hematoxylin and eosin (HE) staining of brain sections revealed severe lymphocyte sequestration and infiltration (Figure 1E), parasitized‐RBCs (pRBCs), and RBCs sequestration (Figure 1F) in the ECM brainstem compared to the healthy brain (Figure 1G). The brainstem is responsible for vital functions such as the cardiovascular and respiratory systems, so we paid more attention to this region in the next study.

### 2.2 ScRNA‐seq revealed CD8^+^ T cell infiltration in the ECM brainstem

**FIGURE 2:**
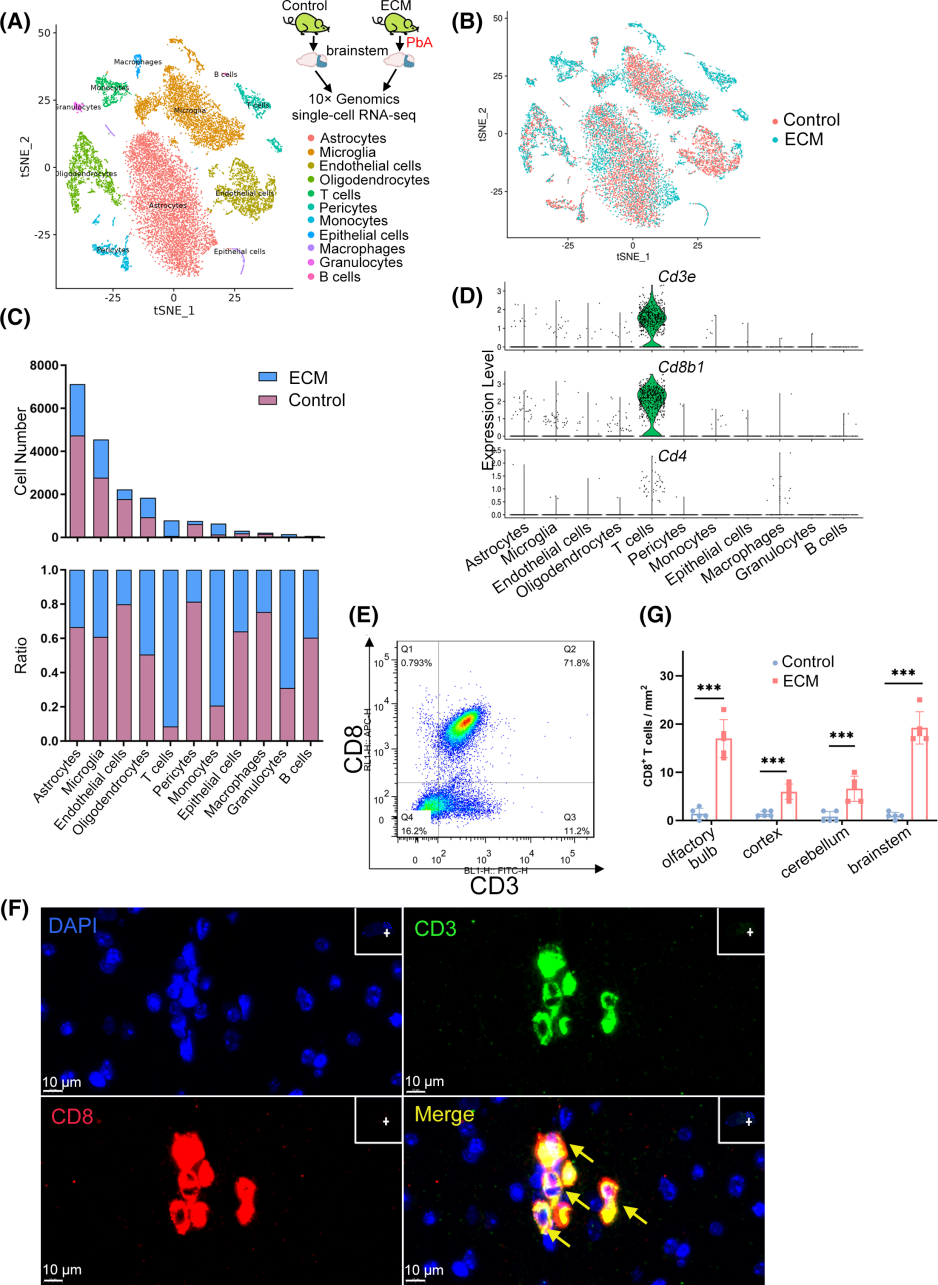
scRNA‐seq reveals CD8^+^ T cell infiltration in the brainstem of mice with experimental cerebral malaria (ECM). (A) Single‐cell RNA‐seq (scRNA‐seq) of the brainstem from ECM and healthy mice using the 10x Genomics Chromium platform. t‐SNE clustering of 18,647 single‐cell transcriptomes (7176 from ECM mice and 11,471 from healthy mice) colored by significant cell‐type clusters. (B) t‐SNE clustering as in (A), but colored by grouping. (C) Comparison of the cell number and the ratio of all cell types between ECM and healthy mice. (D) Violin plot showing the expression of *Cd3e* (encoding CD3), *Cd8b1* (encoding CD8b), and *Cd4* (encoding CD4) in all cell types in the ECM brainstem. Each dot represents the gene expression levels in a single cell. (E) Brain‐infiltrated T cells of the ECM were detected by flow cytometry in three independent experiments. (F) Immunofluorescence staining of CD3^+^CD8^+^ T cells in the ECM brainstem. Green, CD3; red, CD8b; blue, DAPI (nuclei). The yellow arrows indicate CD3 and CD8 double‐positive T cell infiltration in the brainstem. Scale bar, 10 μm. (G) Quantitative results of CD8^+^ T cell infiltration in different brain regions of ECM mice. Data are represented as mean ± SEM, *n* = 5 fields/region, ***p < 0.001.

To explore the cell composition of the brainstem, scRNA‐seq was performed on the brainstem of healthy and ECM mice at 7 dpi using the 10x Genomics platform (Figure 2A). The analysis of 18,647 high‐quality single‐cell transcriptomes (7176 from ECM mice, 11,471 from healthy mice) with t‐stochastic neighbor embedding (t‐SNE) revealed 11 distinct cell types in the brainstem (Figure 2A). Characterization of cell markers identified these cells as astrocytes, microglia, endothelial cells (ECs), oligodendrocytes, T lymphocytes, pericytes, monocytes, epithelial cells, macrophages, granulocytes, and B lymphocytes (Figure S1A). Neurons were not identified, probably because they were too large to be loaded on the platform. Notably, the T lymphocytes were almost exclusively from the ECM brainstems (Figure 2B, Figure S1B), which accounted for 9.95% of the total transcriptome analyzed, while a few T lymphocytes in the healthy brainstem were probably due to inadequate perfusion. ECM was also accompanied by a significantly increased number of monocytes and a decreased number of ECs and pericytes (Figure 2C, Figure S1B), suggesting a serious breakdown of the BBB. ScRNA‐seq also revealed a pro‐inflammatory transcriptomic shift in the ECM brainstem compared with the healthy brainstem (Figure S1C).

The scRNA‐seq data revealed that T cells in the ECM brainstem expressed CD3^+^CD8^+^CD4^−^ T markers (Figure 2D, Figure S1D). To confirm this result, brain‐infiltrated T cells were negatively enriched from the ECM brain with immunomagnetic beads, and flow cytometry assays demonstrated that most of the brain‐infiltrated T cells were CD3^+^CD8^+^ T cells (Figure 2E). Immunofluorescence staining of brain sections also showed that the ECM brainstem was infiltrated by a large number of CD3^+^CD8^+^ T cells (Figure 2F,G, Figure S2A), In comparison, neither CD8^+^ T cells nor other immune cells were observed in the healthy brainstem (Figure S2B).

### 2.3 Brain‐infiltrated CD8^+^ T cells are highly activated and cytotoxic

**FIGURE 3:**
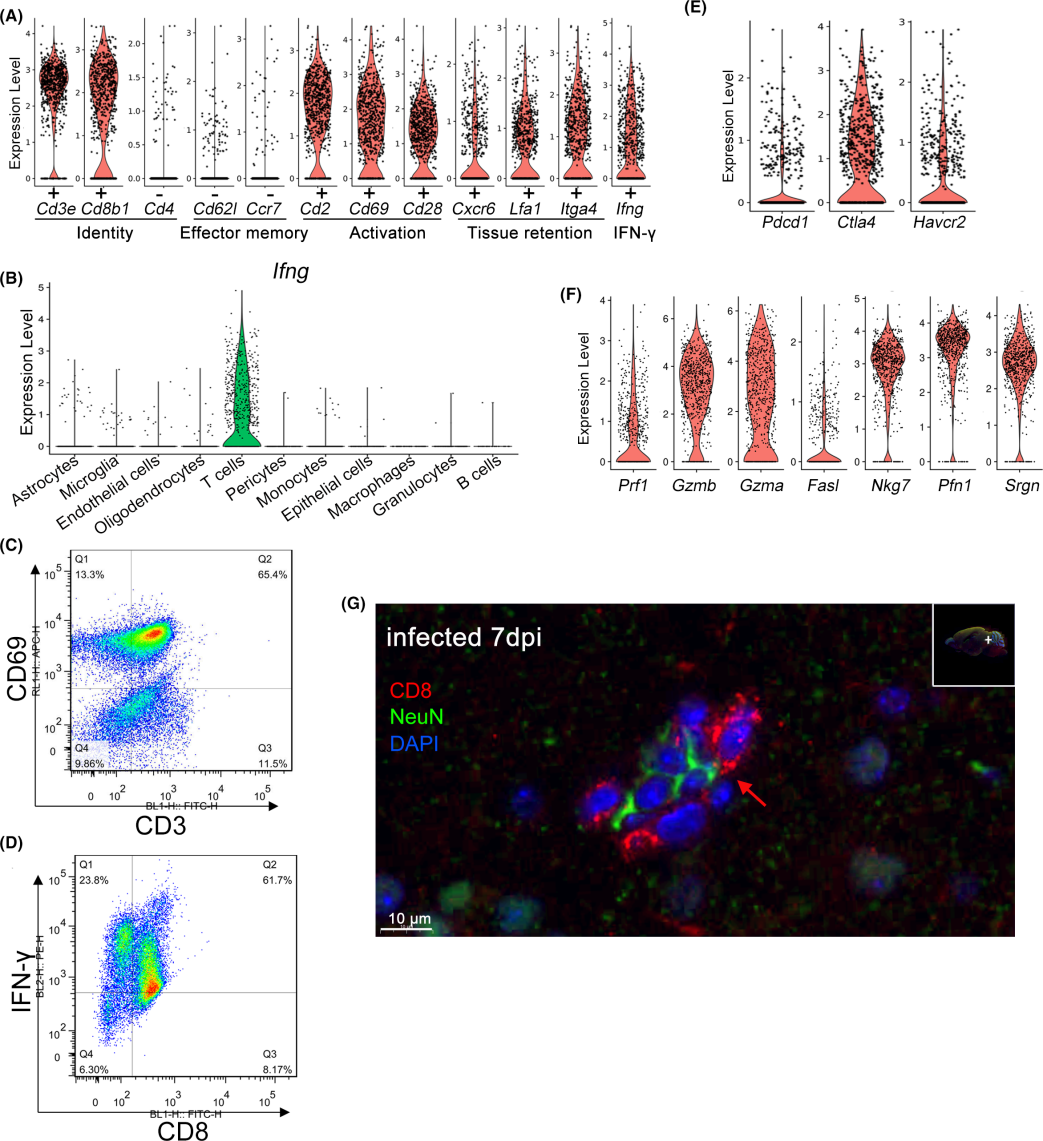
Brain‐infiltrated CD8^+^ T cells were highly activated and cytotoxic. (A) Brain infiltrated CD8^+^ T cells exhibit markers of effector memory T cells (*Cd62l* ^low^, *Ccr7* ^low^), activation (*Cd2* ^high^, *Cd69* ^high^, *Cd28* ^*high*^), tissue residence (*Cxcr6* ^high^, *Lfa1* ^high^, *Itga4* ^high^), and express interferon‐γ (*Ifng*). Each dot represents the expression levels in a single cell, *n* = 714 CD8^+^ T cells from the experimental cerebral malaria (ECM) brainstem. (B) Violin plots showing the gene expression level of *Ifng* (encoding IFN‐γ) in various cell types of the ECM brainstem. Each dot represents the gene expression levels in a single cell. Flow cytometry was used to detect the expression of CD69 (C) and IFN‐γ (D) in brain‐infiltrated T cells, three independent experiments. (E) Violin plots showing the expression of the checkpoint genes *Pdcd1* (encoding PD‐1), *Ctla4* (encoding CTLA‐4), and *Havcr2* (encoding Tim‐3) in various cell types. Each dot represents the gene expression levels in a single cell, *n* = 714 CD8^+^ T cells from the ECM brainstem. (F) Expression of the perforin (*Prf1*), granzyme B (*Gzmb*), granzyme A (*Gzma*), fas ligand (*Fasl*), NKG‐7 (*Nkg7*), profilin‐1 (*Pfn1*), and serglycin (*Srgn*) in brain infiltrated CD8^+^ T cells. Each dot represents the gene expression levels in one cell, *n* = 714 CD8^+^ T cells from the ECM brainstem. (G) Immunofluorescence staining of CD8 and NeuN in the brain sections from ECM brain sections. Red, CD8b (CD8^+^ T cells); green, NeuN (neurons); blue, DAPI (nuclei). The red arrow indicates the colocalization of CD8^+^ T cells and neurons. Scale bar, 10 μm.

The characteristics of the brain‐infiltrated CD8^+^ T cells were analyzed. Brain‐infiltrated T cells expressed markers of effector memory (*Cd62l* ^low^, *Ccr7* ^low^), activation (*Cd2* ^high^, *Cd69* ^high^, *Cd28* ^high^), tissue residence (*Cxcr6* ^high^, *Lfa1* ^high^, *Itga4* ^high^), and pro‐inflammatory cytokines (*Ifng* ^high^) (Figure 3A). Brain‐infiltrated CD8^+^ T cells were the primary source of interferon‐γ (IFN‐γ) detected in this scRNA‐seq dataset (Figure 3B). Flow cytometry confirmed that brain‐infiltrated T cells exhibited CD69^high^ and IFN‐γ^high^ phenotypes (Figure 3C,D), although the results of flow cytometry were not the same as those of single‐cell analysis. Brain‐infiltrated T cells showed a high expression of immune checkpoint molecules, including PD‐1 (encoded by *Pdcd1*), CTLA‐4 (encoded by *Ctla4*), and TIM‐3 (encoded by *Havcr2*) (Figure 3E).

Brain‐infiltrated CD8^+^ T cells exhibited strong potential cytotoxic activity, as evidenced by high expression of perforin, granzyme B, granzyme A, fas ligand, NKG‐7 (natural killer cell group 7), profilin‐1, and serglycin (encoded by *Prf1*, *Gzmb*, *Gzma*, *Fasl*, *Nkg7*, *Pfn1*, and *Srgn*, respectively) (Figure 3F). Immunofluorescence staining of the ECM brain section showed that brain‐infiltrated CD8^+^ T cells were in close proximity to neurons (Figure 3G). Considering the cytotoxicity of these CD8^+^ T cells, ECM would likely give rise to autonomic dysfunction due to neuronal damage. Our previous work also showed that brain‐infiltrated T cells could kill ECs,^23^ further aggravating the BBB breakdown and infiltration of T cells during ECM.

### 2.4 Brain‐infiltrated CD8^+^ T cells are antigen‐experienced and show proliferative activity

**FIGURE 4:**
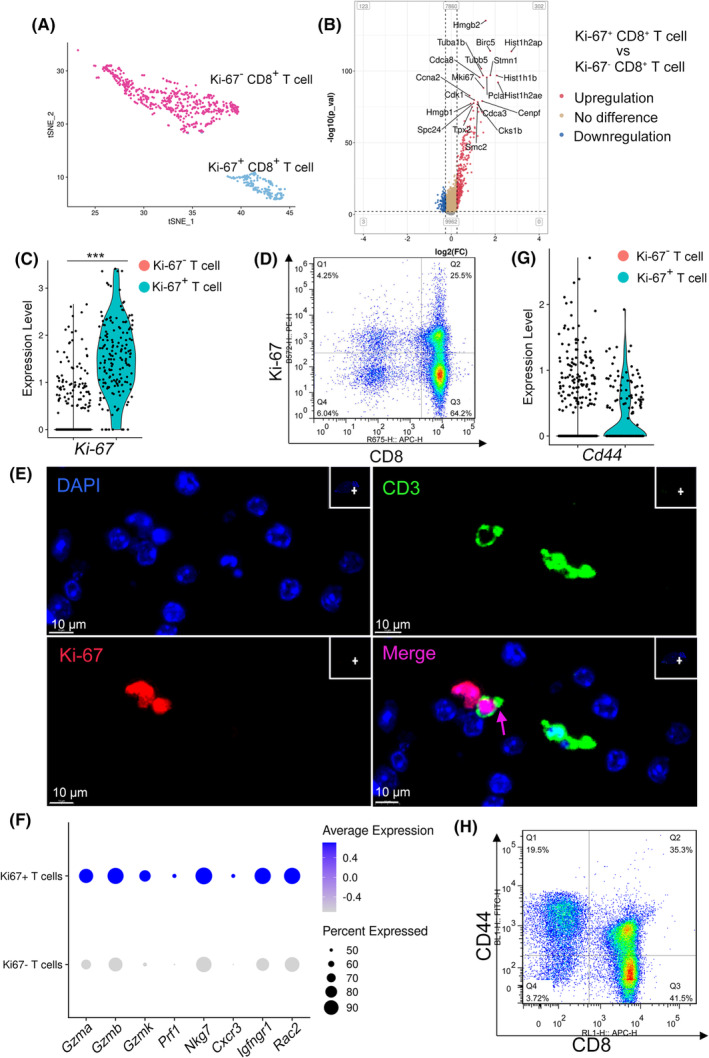
Brain‐infiltrated CD8^+^ T cells were antigen‐experienced. (A) t‐SNE plot of 714 brain‐infiltrated CD8^+^ T cells transcriptomes, colored by Ki‐67 (Ki‐67^−^, purple; Ki‐67^+^, blue), *n* = 540 Ki‐67^−^CD8^+^ T cells, *n* = 174 Ki‐67^−^CD8^+^ T cells from the experimental cerebral malaria (ECM) brainstem. Each dot represents a single T cell transcriptome. (B) Volcano plots showing the top 20 differentially expressed genes between Ki‐67 positive and Ki‐67 negative CD8^+^ T cells from the ECM brainstem. Each dot represents an individual gene. (C) Violin plots showing the expression of *Ki‐67* in Ki‐67 positive and Ki‐67 negative CD8^+^ T cells from the ECM brainstem. Each dot represents the gene expression levels in a single cell. ***p < 0.001, *n* = 714 CD8^+^ T cells from the ECM brainstem. (D) Flow cytometry showing the expression of Ki‐67 in brain‐infiltrated T cells, three independent experiments. (E) Immunofluorescence staining of Ki‐67^+^ T cells in the ECM brainstem. Green, CD3; Red, Ki‐67; blue, DAPI (nuclei). Purple arrow indicate Ki‐67 and CD3 double‐positive cell infiltration in the brainstem. Scale bar, 10 μm. (F) Dot plot of the activation and functional genes (*Gzma*, *Gzmb*, *Gzmk*, *Prf1*, *Nkg7*, *Cxcr3*, *Ifngr1*, and *Rac2*) expression between Ki‐67 positive and Ki‐67 negative CD8^+^ T cells from ECM brainstem. (G) Violin plots showing the expression of *Cd44* in Ki‐67 positive and Ki‐67 negative CD8^+^ T cells from the ECM brainstem, *n* = 714 CD8^+^ T cells from the ECM brainstem. Each dot represents the gene expression levels in a single cell. (H) Flow cytometry showing the expression of CD44 in brain‐infiltrated T cells from the ECM brainstem, three independent experiments.

Brain‐infiltrated CD8^+^ T cells can be divided into two distinct subgroups (Figure 4A, Figure S3), which exhibit significant differences at the transcriptional level (Figure 4B). We defined these cells as Ki‐67^+^ CD8^+^ T cells and Ki‐67^−^ CD8^+^ T cells (Figure 4A,C). By analyzing the top 20 differentially expressed genes between these two CD8^+^ T cell subsets, we found that most of the differentially expressed genes were related to DNA replication, transcription, cell cycle, and cell division (Figure 4B), indicating that Ki‐67^+^ CD8^+^ T cells may represent a subgroup of dividing cells. Flow cytometry (Figure 4D) and immunofluorescence staining of brain sections (Figure 4E) also demonstrated many Ki‐67 positive cells in the brain‐infiltrated CD8^+^ T cells in the brainstem, although the results were not identical to those of single‐cell analysis.

Compared with Ki‐67^−^ CD8^+^ T cells, Ki‐67^+^ CD8^+^ T cells have a higher transcriptional level of genes related to T cell function and activation (*Gzma*, *Gzmb*, *Gzmk*, *Prf1*, *Nkg7*, *Cxcr3*, *Ifngr1*, and *Rac2*) (Figure 4F), suggesting that Ki‐67^+^ CD8^+^ T cells have a higher degree of activation and stronger cytotoxicity to ECs and brain parenchyma cells. We noted that CD44, a marker for antigen‐experienced T cells, was more prevalent in Ki‐67^+^ CD8^+^ cells than in Ki‐67^−^ CD8^+^ T cells (37.4% vs. 23.5%) (Figure 4G). Brain‐infiltrated T cells were negatively enriched, and CD44 was detected by flow cytometry. Based on this analysis, CD44‐positive cells account for 35% of the total brain‐infiltrated CD8^+^ T cells, which is consistent with our scRNA‐seq result (Figure 4H). Together, the proliferation state of Ki‐67^+^ CD8^+^ T cells indicated that T cells migrate to the brain after being activated in the spleen, then are re‐activated in situ in an antigen‐dependent manner, and brain parenchyma cells may play the role of antigen‐presenting cells (APCs).

### 2.5 Brain parenchyma cells are highly responsive to interferon signaling

**FIGURE 5:**
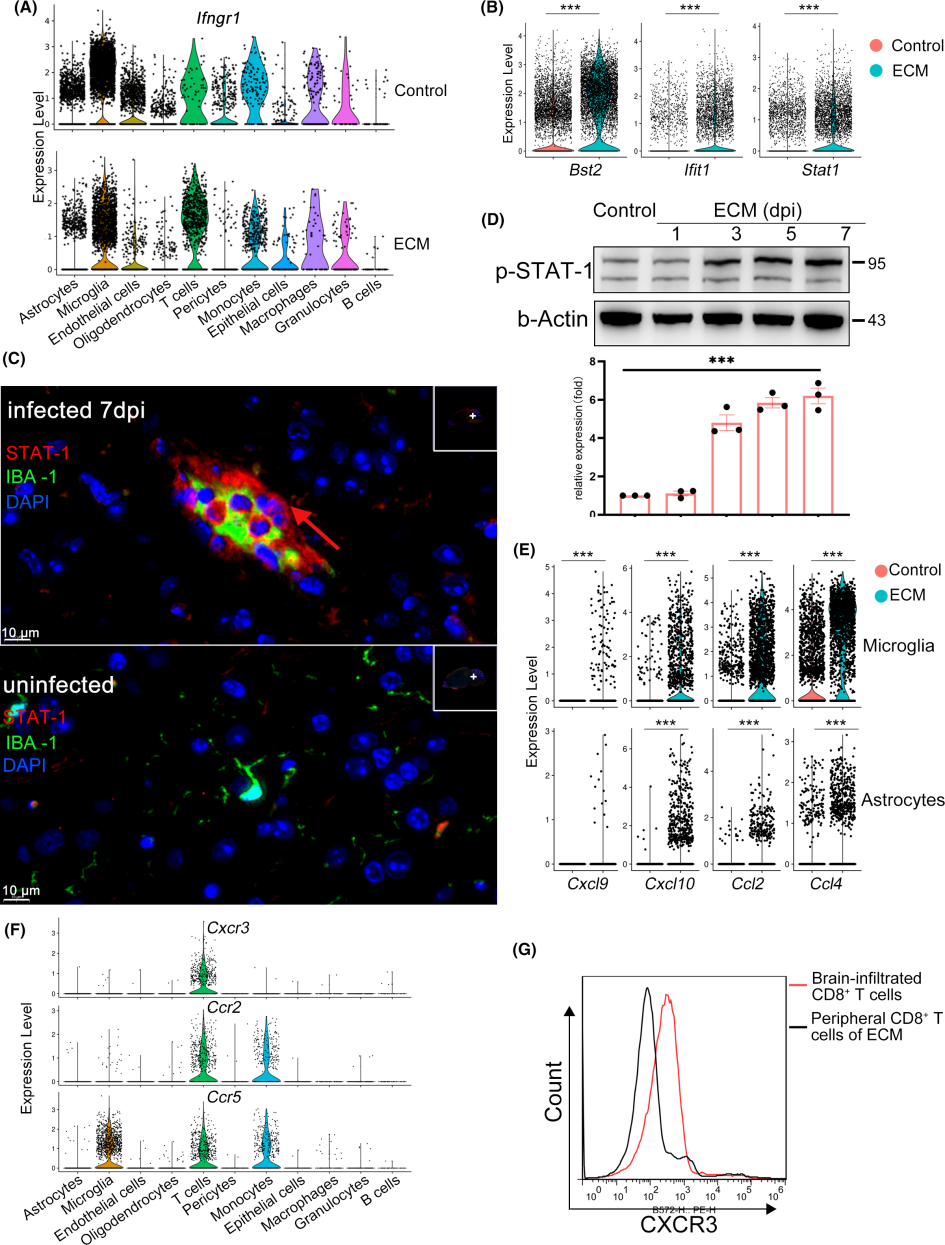
Brainstem parenchyma cells responded to interferon signaling in experimental cerebral malaria (ECM) mice. (A) Violin plots showing the expression of IFN‐γ receptor 1 (*Ifngr1*) in various brain parenchyma cell types both in the ECM and healthy mice. Each dot represents the gene expression levels in a single cell. (B) Expression of the *Bst2*, *Ifit1*, and *Stat1* in the ECM brainstem and healthy brainstem. Each dot represents the gene expression levels in a single cell. ***p < 0.001. (C) Immunofluorescence staining of STAT‐1 and IBA‐1 in ECM brainstem and healthy brainstem. Red, STAT‐1; green, IBA‐1; blue, nuclei. The red arrow indicates STAT1‐positive microglia in the ECM brainstem. Scale bar, 10 μm. (D) Western blot results of p‐STAT‐1 from the ECM brainstem; samples were taken from ECM mice at 1, 3, 5, and 7 dpi. Data are mean ± SEM. Each dot represents one replicate, *n* = 3, **p < 0.01. (E) Expression of the chemokines *Cxcl9*, *Cxcl10*, *Ccl2*, and *Ccl4* in microglia and astrocytes from ECM mice and healthy mice. Each dot represents the gene expression levels in a single cell. ***p < 0.001. (F) Expression of the chemokine receptors *Cxcr3*, *Ccr2*, and *Ccr5* in various cell types from ECM brainstem parenchyma cells. Each dot represents the gene expression levels in a single cell. (G) Flow cytometry showing the expression of CXCR3 in brain‐infiltrated T cells and peripheral CD8^+^ T cells in ECM mice, three independent experiments.

After identifying the profile of brain‐infiltrated CD8^+^ T cells in the ECM brainstem, we were interested in the interactions between brain‐infiltrated CD8^+^ T cells and brain parenchyma cells during neuroinflammation. The activation of parenchyma cells is a common response to neuroinflammation, in which IFN‐γ plays an extremely important role. Brain‐infiltrated CD8^+^ T cells were the most notable source of IFN‐γ in the ECM brainstem (Figure 3B, Figure S4A). ScRNA‐seq analysis demonstrated that although a variety of brain parenchyma cells (including astrocytes, microglia, and ECs), both from healthy and ECM, showed high transcription of IFN‐γ receptor (*Ifngr1*, *Ifngr2*) (Figure 5A, Figure S4B), upregulated transcription of molecules downstream of the IFN‐γ pathway (*Ifit1*, *Stat1*, *and Bst2*) mainly occurred in the brainstem of ECM mice (Figure 5B, Figure S4C). Immunofluorescence staining revealed strongly increased staining of STAT1, a protein that is upregulated upon interferon signaling, in microglia of the ECM brainstem compared to microglia of the healthy brainstem (Figure 5C, Figure S4D). STAT‐1 phosphorylation was also upregulated with disease progression (Figure 5D). These results demonstrated that brain parenchyma cells experience a strong response to IFN‐γ stimulation.

Once activated by IFN‐γ, brain parenchyma cells, especially astrocytes and microglia, may secrete T cell chemokines CXCL‐10, CCL‐2, and CCL‐4 (Figure 5E, Figure S5A), and brain‐infiltrated CD8^+^ T cells expressed their corresponding receptors CXCR3, CCR2, and CCR5 (Figure 5F, Figure S5B), which could recruit CD8^+^ T cells to migrate into the brain in a chemokine‐dependent manner, further aggravating the pro‐inflammatory state in the brainstem. To further expand on this finding, we compared CXCR3 and CCR2 expression between brain‐infiltrated CD8^+^ T cells and peripheral CD8^+^ T cells in ECM mice by flow cytometry, brain‐infiltrated CD8^+^ T showed higher chemokine receptor CXCR3 and CCR2 expression than peripheral CD8^+^ T cells (Figure 5G, Figure S5C).

### 2.6 Activated astrocytes may act as cross‐presenting cells during ECM

**FIGURE 6:**
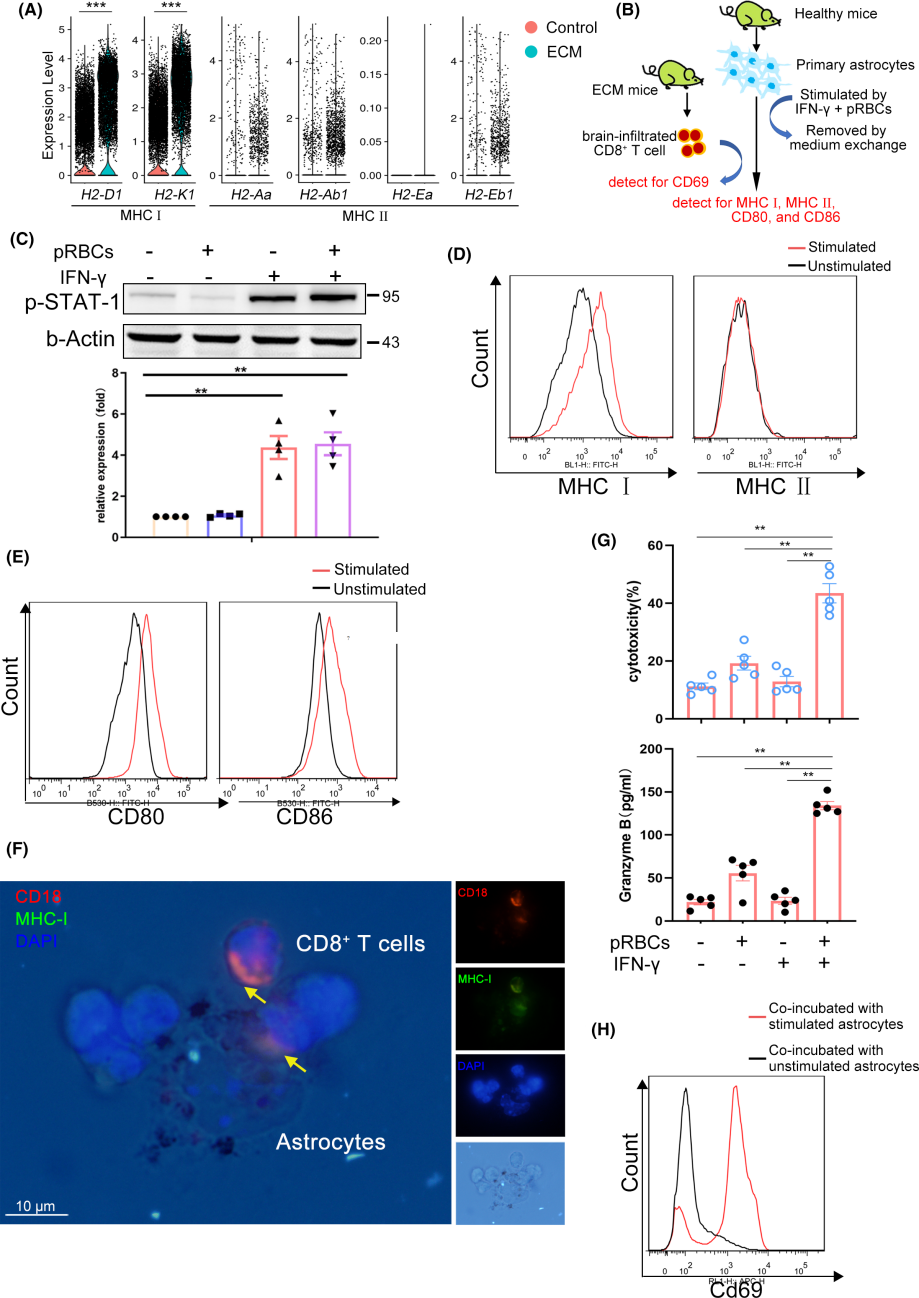
Activated astrocytes may act as cross‐presenting cells to promote CD8^+^ T cell activation. **(A)** Violin plots showing the expression of MHC I (*H2‐D1*, *H2‐K1*) and MHC II (*H2‐Aa/Ab*, *H2‐Ea/Eb*) in the experimental cerebral malaria brainstem and healthy brainstem. Each dot represents the gene expression levels in a single cell. ***p < 0.001. (B) Schematic diagram of astrocytes and CD8^+^ T cell cocultured model. (C) Western blot results of phosphorylation of STAT‐1 after stimulation or unstimulation with IFN‐γ and pRBCs. Data are mean ± SEM. Each dot represents one replicate, *n* = 4, **p < 0.01. Flow cytometry results of MHC I, MHC II (D), CD80, and CD86 (E) expression in primary astrocytes after being stimulated with IFN‐γ and pRBCs, three independent experiments. (F) Immunofluorescence of MHC I and LFA showed that CD8^+^ T cells and astrocytes formed an “immune synapse”‐like structure, Green, MHC I; Red, LFA‐β subunit (CD18); blue, DAPI (nuclei). The yellow arrow indicates the colocalization of MHC I and LFA. Scale bar, 10 μm. (G) Cytotoxicity of splenic CD8^+^ T cells to activated astrocytes was evaluated by lactate dehydrogenase content (upper). Granzyme B content was detected by ELISA (down). Data are mean ± SEM, each dot represents one replicate, *n* = 5, **p < 0.01. (H) Flow cytometry result of CD69 expression in CD8^+^ T cells coincubated with stimulated or unstimulated astrocytes, three independent experiments.

Activated CD8^+^ T cells are re‐stimulated by brain parenchyma APCs in the brain after infiltration into the brainstem. We found that brain parenchyma cells, such as astrocytes, microglia, and ECs, had markedly upregulated antigen‐presenting molecules, especially major histocompatibility complex I (MHC I) (H‐2K^b^ and H‐2D^b^) during infection, while MHC II (I‐A^b^ and I‐E^b^) was undetectable during infection (Figure 6A, Figure S6). Studies have shown that microglia could act as cross‐presenting cells to promote brain‐infiltrated CD8^+^ T cell activation,^24^ but little is known about astrocytes. A coculture model was established, in which primary astrocytes were treated or untreated with pRBCs and IFN‐γ to mimic the pro‐inflammatory microenvironment observed in the ECM brain, and then coincubated with brain‐infiltrated CD8^+^ T cells from ECM mice (Figure 6B). As a marker of activated IFN‐γ signaling, expression, and phosphorylation of STAT‐1 were upregulated in astrocytes after stimulation according to western blotting (Figure 6C). Flow cytometry analysis indicated that the pro‐inflammatory microenvironment upregulated the expression of MHC I but not MHC II in astrocytes (Figure 6D), as well as costimulatory molecules, CD80 and CD86 (Figure 6E). Immunofluorescence of MHC I and LFA1 (CD18) showed that CD8^+^ T cells and astrocytes formed an “immune synapse”‐like structure (Figure 6F), A lactate dehydrogenase (LDH) cytotoxicity assay revealed that astrocytes could be killed by CD8^+^ T cells in a granzyme B‐dependent manner (Figure 6G). CD8^+^ T cells could be re‐stimulated by activated astrocytes, as CD69 levels were higher when coincubated with activated astrocytes (Figure 6H). These results suggest that brain parenchyma cells, such as astrocytes, could cross‐present parasite antigens to CD8^+^ T cells after IFN‐γ stimulation.

### 2.7 Activated CD8^+^ T cells and PD‐1 signaling are therapeutic targets for CM

**FIGURE 7:**
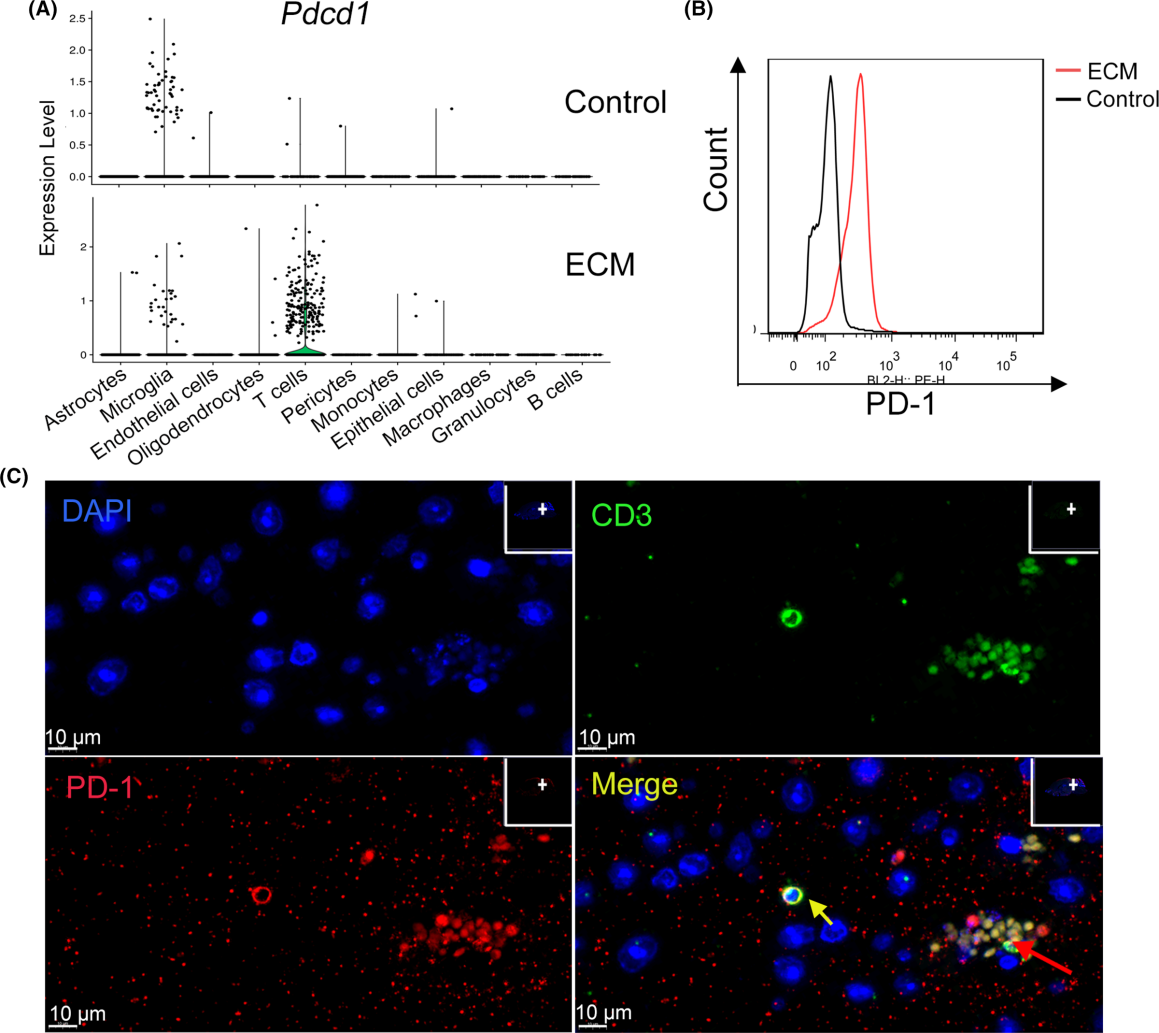
Brain‐infiltrated CD8^+^ T are PD‐1 positive. (A) Violin plots showing the expression of *Pdcd1* (encoding PD‐1) in the experimental cerebral malaria (ECM) brainstem and healthy brainstem. Each dot represents the gene expression levels in a single cell. (B) Flow cytometry of PD‐1 expression on peripheral CD8^+^ T cells of ECM mice and healthy mice, three independent experiments. (C) Immunofluorescence staining of PD‐1^+^ T cells in the ECM brainstem. Green, CD3; red, PD‐1; blue, DAPI (nuclei). The yellow arrows indicate PD‐1^+^ T cell infiltration in the brainstem, and the red arrows indicate sequestration of RBCs and pRBCs. Scale bar, 10 μm.

Brain‐infiltrated CD8^+^ T cells expressed high levels of immune checkpoint molecules within 1 week following PbA infection (Figures 3B and 7A), including PD‐1. Flow cytometry analysis also revealed that PD‐1 expression is upregulated in peripheral CD8^+^ T cells of ECM mice compared to healthy mice (Figure 7B). An immunofluorescence assay confirmed that PD‐1^+^ T cell infiltration was accompanied by severe BBB breakdown and pRBC sequestration (Figure 7C).

**FIGURE 8:**
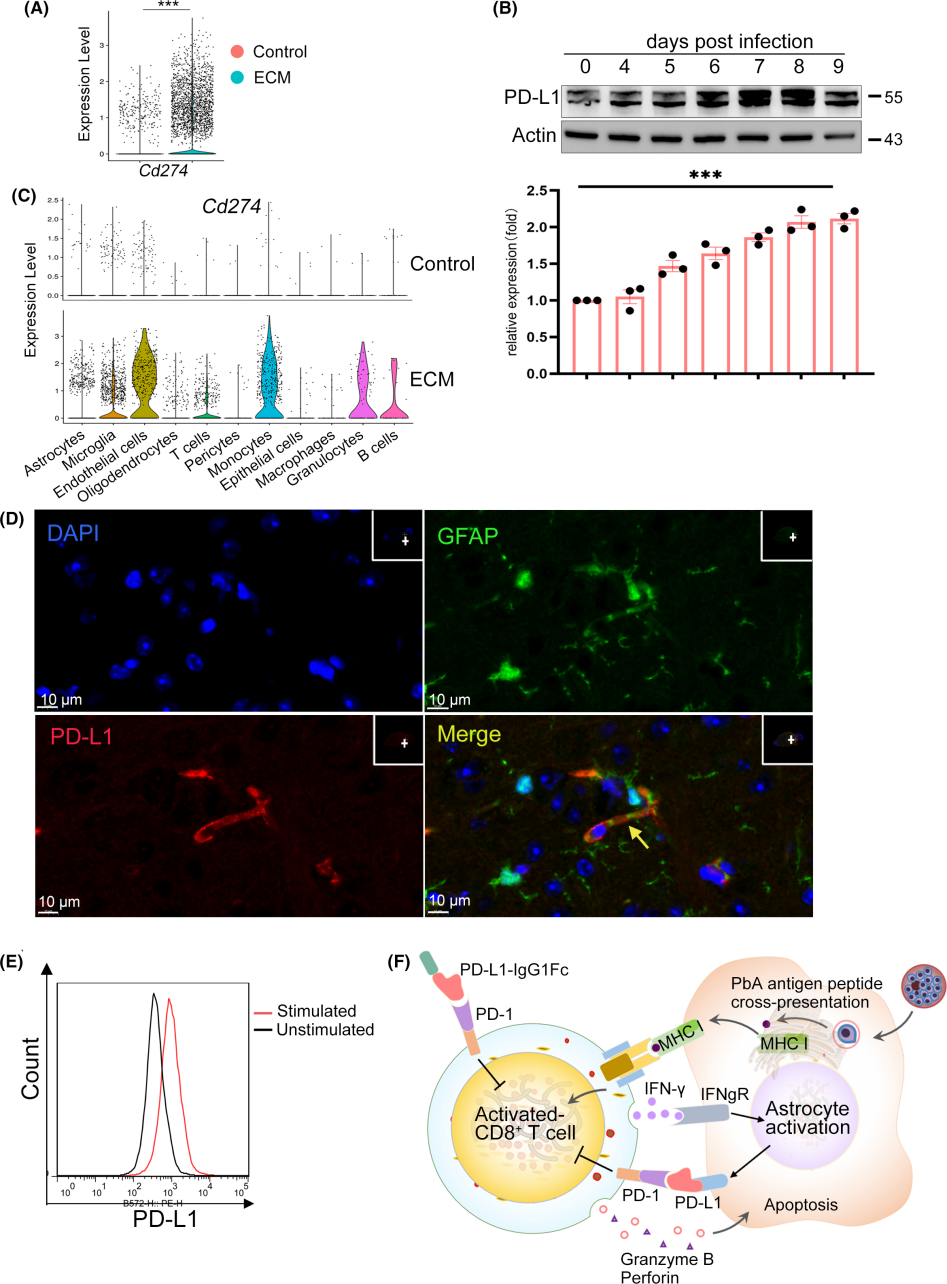
Activated CD8^+^ T cells and the PD‐1 signal pathway can be used as therapeutic targets for experimental cerebral malaria (ECM) treatment. (A) Violin plots showing the expression of *Cd274* (encoding PD‐L1) in the ECM and healthy brainstem. Each dot represents the gene expression levels in a single cell. ***p < 0.001. (B) Western blot results of PD‐L1 expression from the ECM brainstem after 4–9 dpi. Data are mean ± SEM. Each dot represents one replicate, *n* = 3, ***p < 0.001. (C) Violin plots showing the expression of *Cd274* in various cell types of the ECM brainstem and healthy brainstem. Each dot represents the gene expression levels in a single cell. (D) Immunofluorescence staining of PD‐L1^+^ astrocytes in the ECM brainstem. Green, GFAP; red, PD‐L1; blue, DAPI (nuclei). The yellow arrows indicate PD‐L1^+^ astrocytes in the brainstem. Scale bar, 10 μm. (E) Flow cytometry results of PD‐L1 expression in astrocytes after IFN‐γ and pRBCs stimulation in Figure 6B, three independent experiments. (F) PD‐L1 fusion protein could be used as an immunotherapy strategy for ECM by downregulating the over‐activation of CD8^+^ T cells.

Therefore, we next investigated the transcription of the PD‐1 ligand‐PD‐L1 (encoded by *Cd274*) in ECM brainstem cells. According to scRNA‐seq analysis, PD‐L1 was significantly upregulated in the brainstem within 1 week following PbA infection (Figure 8A). Western blotting revealed increased PD‐L1 expression in the brainstem of ECM mice with the progression of the disease (Figure 8B). A basal level of PD‐L1 was expressed in approximately 2.8% of microglia in healthy mice, but the induced expression was detectable in 32.5% of cells within 1 week (Figure 8C). Immunofluorescence assays confirmed that PD‐L1 was upregulated in astrocytes during PbA infection (Figure 8D). In vitro experiments demonstrated that astrocyte PD‐L1 was upregulated after IFN‐γ stimulation detected by flow cytometry (Figure 8E). These results suggest that brain parenchyma cells have the potential to inhibit brain‐infiltrated CD8^+^ T cell activation through the PD‐1/PD‐L1 negative pathway (Figure 8G).

## 3 DISCUSSION

Cerebral malaria is an acute, lethal neurological complication caused by *P.f* infection, and extensive evidence has confirmed that immune cells mediate this disease.^25^ CD4^+^ and CD8^+^ T cells, γδ T cells, natural killer cells (NK), NKT cells, neutrophils, and macrophages can adhere or sequester to the brain microvessels in ECM mice^6^, ^26^, ^27^, ^28^ and children with CM,^14^ but CD8^+^ T cells play an irreplaceable role in the pathogenesis of CM. A comprehensive profile of brain‐infiltrated CD8^+^ T cells and their interactions with brain parenchyma cells in the ECM remains elusive.

Damage to the CNS is the main cause of death in patients and mice with CM. Our study showed the damage to the CNS was quite inconsistent, compared with the cortex or cerebellum, the olfactory bulb, and the brainstem showed the most severe cell apoptosis and immunopathology. This phenomenon is consistent with existing research.^11^, ^29^ Brain‐infiltrated CD8^+^ T cells were highly activated and showed strong cytotoxicity during ECM.^30^ Considering the extensive CD8^+^ T cell infiltration and its colocation with neurons, it is reasonable to speculate that brain‐infiltrated CD8^+^ T cells are the main contributors to CNS damage. The brainstem controls vital functions such as the cardiovascular and respiratory systems; it is likely that mice succumb to ECM due to the widespread CNS damage observed in this region. As evidence, Taylor et al.^5^ point out that the most likely cause of human CM death was brainstem herniation.

The pathogenesis of ECM is accompanied by severe cytokine release syndrome (CRS) and brain inflammation, in which IFN‐γ plays an extremely important and essential role.^31^, ^32^ In the present study, the scRNA‐seq analysis revealed that almost all IFN‐γ‐positive cells in the ECM brainstem were brain‐infiltrated CD8^+^ T cells, while healthy mice had few IFN‐γ‐positive cells. CD8^+^ T cells play essential roles in protection from CNS infection, but infiltrated CD8^+^ T cells induce immunopathology during neuroinflammation caused by infection.^33^, ^34^ Therefore, it is necessary to maintain a delicate balance between pro‐and anti‐inflammatory immune responses to control parasitemia without inducing immunopathology.^35^

Brain‐infiltrated CD8^+^ T cells are heterogeneous and can be divided into two subgroups: Ki‐67^+^ and Ki‐67^−^ CD8^+^ T cells. Ki‐67 is a well‐known cell‐cycle‐associated antigen for the evaluation of cell proliferation,^36^, ^37^ and CD44 is a marker of antigen‐experienced T cells.^38^, ^39^ During PbA infection, CD8^+^ T cells were first activated at the site of the spleen or lymph node, followed by infiltration, re‐activation, and proliferation in the brain in an antigen‐specific and chemokine‐dependent manner. The present transcriptome analysis has shown that Ki‐67^+^ CD8^+^ T cells express molecules associated with cell cycling and activation, and may represent a subtype of proliferating CD8^+^ T cells that are re‐activated in situ in the brainstem.^16^, ^40^ It would certainly be interesting to identify a new subtype of CD8^+^ T cells or assess the pathogenic (maybe some protective effect) role of *Plasmodium*‐specific brain‐infiltrated CD8^+^ T cells play.

Antigen‐presenting cells situated in the CNS are thought to mediate CD8^+^ T cell entry into the parenchyma during neuroinflammation. As CNS‐resident cells, astrocytes and microglia are extremely sensitive to brain injury and function as a bridge *linking* the CNS and the immune system during neuroinflammation.^15^, ^41^ Traditionally, as exogenous antigen, PbA antigens should be presented to CD4^+^ T cells by APCs in an MHC II‐dependent manner. Howland et al. and Swanson et al.^11^, ^31^ showed that ECs can internalize PbA antigen and cross‐present it to activated CD8^+^ T cells in an MHC I‐dependent manner after stimulation with IFN‐γ. In the present study, we demonstrated that astrocytes may also cross‐present exogenous antigens to CD8^+^ T cells in the ECM, which confirms the findings in other models.^42^ Chemokines and chemokine receptors are well‐known regulators of leukocyte migration, and it is possible that brain parenchyma cell‐secreted chemokines (CCL‐2, CCL‐4, and CXCL‐10) and their corresponding chemokine receptors (CCR2, CCR5, and CXCR3), which are highly expressed on activated T cells, play an essential role in this process. Brain parenchyma cells and infiltrated CD8^+^ T cells may communicate with and activate each other,^43^, ^44^ further exacerbating the symptoms of ECM. Importantly, it has been reported that CXCL‐10 may act as a cue for growth acceleration in malaria parasites.^45^ However, these results still could not explain why only CD8^+^ T cells infiltrated the brain parenchyma in the ECM, but not CD4^+^ T cells or B cells, which is consistent with the existing research results.^46^

Since brain‐infiltrated CD8^+^ T cells are the main effectors leading to CNS injury, a treatment that targets over‐activated CD8^+^ T cells could be a promising therapeutic strategy for CM. Adjunctive therapy for ECM was supported by a series of studies that the blocked vascular adhesion of T cells to the brain vasculature using anti‐CD146, anti‐LFA‐1, or anti‐VLA‐4 antibodies, respectively, thus rescuing mice from late‐stage ECM.^11^, ^47^, ^48^ However, antibody treatments targeting adhesion molecules may have unpredictable side effects, and functional regulation of CD8^+^ T cells by immune checkpoint molecules is a promising alternative therapeutic strategy for CM that has been used successfully in clinical applications. In the present study, PD‐L1 upregulation in glial cells occurred as a response to IFN‐γ produced infiltrated PD‐1 high CD8^+^ T cells during PbA infection. According to the scRNA‐seq results, the number of PD‐L1‐positive cells increased by more than ten‐fold in the ECM brainstem compared to the healthy brainstem. The upregulation of PD‐L1 limits CNS pathology through the suppression of pro‐inflammatory cytokine production and cytotoxicity by brain‐infiltrated CD8^+^ T cells. Blocking the interactions in the PD‐1/PD‐L1 pathway between CD8^+^ T cells and either astrocytes or microglia resulted in increased IFN‐γ and IL‐2 production in murine cytomegalovirus‐induced encephalitis.^20^ Similarly, increased IFN‐γ production by T cells has been observed in PD‐L1 knockout mice,^22^ indicating a downregulatory role of PD‐L1.

Brain parenchyma cells have the potential to inhibit the over‐activation of CD8^+^ T cells through PD‐L1, but they still cannot inhibit the serious consequences caused by ECM. It is possible that the inflammatory response and immunopathology caused by CD8^+^ T cells are too strong, or a negative feedback pathway was initiated either too late or not strong enough to inhibit neuroinflammation in the brain. Our previous results confirmed that exogenous PD‐L1 fusion protein (PDL1‐IgG1Fc) can reduce the activation and cytotoxicity of CD8^+^ T cells, thus alleviating the neuroinflammation and damage to the CNS caused by ECM.^23^, ^49^ The PD‐L1 fusion protein is thus a promising immunotherapy strategy for CM by downregulating the over‐activation of CD8^+^ T cells (Figure 8F). The PD‐L1 fusion protein may also be used as a novel T‐cell inhibitor for the treatment of diseases caused by abnormal activation of T cells, such as autoimmune diseases.

Although the present study adds to our understanding of CD8^+^ T cells involved in neuroinflammation and immunopathology in CM, there are some limitations. First, neurons were not identified by our scRNA‐seq, probably because they were too large to be loaded,^50^, ^51^ and this analysis, therefore, lacked data on the relationship between neurons and brain‐infiltrated CD8^+^ T cells. Second, the specific mechanism of CD8^+^ T cell infiltration into the brain parenchyma should be studied further to explain why only CD8^+^ T cells, not other immune cells, infiltrate the brain parenchyma during ECM. Antigen presentation or chemokines may explain part of the reason, but it is still far from enough. Third, a control group of mice infected with non‐ECM‐induced *Plasmodium* lacked, it would be interesting to explore the cell composition and pathological changes of the brain in non‐ECM mice.

The present study found CD8^+^ T cell infiltration into the brainstem parenchyma and confirmed their characteristics of continuously activated proliferation during the PbA‐infected ECM model. More importantly, we also demonstrated that astrocytes could act as antigen‐presenting cells to re‐activate brain‐infiltrated CD8^+^ T cells by the antigen cross‐presentation pathway, which appeared to be detrimental to the brain parenchyma cells, both in vitro and in vivo. In the following studies, we will continue to focus on the more in‐depth classification and characterization research of the brain‐infiltrated CD8^+^ T cells. In addition, the interactions between the brain‐infiltrated CD8^+^ T cells and other brain parenchyma cells, such as neurons and astrocytes, may provide new ideas for exploring the pathogenesis of CM and its adjunctive therapy.

## 4 MATERIALS AND METHODS

### 4.1 Ethics statement

All animal experiments were approved by the Institutional Review Board of the Fourth Military Medical University (FMMU) (No:20200407). All efforts were made to minimize the suffering of the animals employed in this study.

### 4.2 Mice and parasites

C57BL/6 male mice (6–8 weeks old) were used for infection experiments; all mice were bred and housed under specific pathogen‐free conditions in the animal center of FMMU. PbA was maintained and used as previously reported in our laboratory. Mice were infected intraperitoneally (i.p.) with 5 × 10^6^ parasitized RBCs (pRBCs) or equal volumes of saline as a control. Peripheral blood parasitemia was determined by Giemsa‐stained, thin blood smears. Mice were monitored daily for symptoms of ECM. We chose 7 days post‐infection (dpi) as the peak of ECM symptoms. All mice were randomly assigned to different groups.

### 4.3 Antibodies and reagents

Antibodies against mouse PD‐L1 (Cat.66248), CD8a (Cat.65069), NeuN (Cat.26975), CCL‐2 (Cat.25542), CD18 (Cat.10554), and STAT‐1 (Cat.10144) were purchased from Proteintech. Antibodies against mouse p‐STAT‐1 (Cat.ET1611) and MHC I (Cat.EM1801) were purchased from HUABIO. Antibodies against mouse IBA‐1 (Cat.ab283346), CD3 (Cat.ab11089), CCR2 (Cat.ab273050), Ki‐67 (Cat.ab15580), PD‐1 (Cat.ab237728) were purchased from Abcam. FITC‐labeled antibodies against mouse CD3 (Cat.100203), CD8a (Cat.100706), CD44 (Cat.103005), CD80 (Cat.104705), CD86 (Cat.105005), MHC I (Cat.114605), MHC II (Cat.109905), APC‐labeled antibodies against mouse CD8a (Cat.100712), CD69 (Cat.104513), PE‐labeled antibodies against mouse Ki‐67 (Cat.652403), CCR2 (Cat.150609), IFN‐γ (Cat.505808), PD‐1 (Cat.621607), CXCR3 (Cat.155903), and PD‐L1 (Cat. 124307) were purchased from Biolegend. ELISA kits for mouse IL‐1β (Cat.1210122), TNF‐α (Cat.1217202), IFN‐γ (Cat.1210002), IL‐10 (Cat.1211002), IL‐6 (Cat.1210602), and TGF‐β (Cat.1217102) were purchased from DAKEWE. Recombinant mouse IFN‐γ (Cat.50709) was purchased from SinoBiological. LDH Cytotoxicity Assay Kit was purchased from Beyotime Biotechnology (Cat.C0017). In Situ Cell Apoptosis Detection Kit (Cat.E607178) was purchased from Sangon. Poly‐L‐lysine was purchased from Sigma‐Aldrich (Cat.P4832). All these antibodies and reagents were used in the schedules and doses indicated.

### 4.4 Single‐cell RNA‐sequencing from ECM and healthy brainstem

We performed scRNA‐seq of all live cells in the brainstem. To this end, healthy and symptomatic ECM mice at 7 dpi were used. Mice were anesthetized and perfused intracardially with saline to remove RBCs and leukocytes from the brain; brainstems were immediately collected. Separate single brainstem cells according to the instructions. Brainstem cells of 3 ECM mice and 3 healthy mice were combined into 1 group, respectively, defined as the ECM group and the control or healthy group. Single‐cell suspensions were loaded with 10x chromium according to the manufacturer's instructions for the 10X Genomics Chromium Single‐Cell 3′ kit (V3). The following cDNA amplification and library construction steps were performed according to the standard protocol. Libraries were sequenced on an Illumina NovaSeq 6000 sequencing system (paired‐end multiplexing run, 150 bp) by LC‐Bio Technology co. Ltd. at a minimum depth of 20,000 reads per cell. Cells were removed if they expressed fewer than 500 unique genes or greater than 25% mitochondrial reads. Our study includes 18,647 cells, with 7176 cells from ECM mice and 11,471 cells from healthy mice. Cell types were determined using a combination of marker genes identified from the literature and a gene ontology for cell types using the web‐based tool (http://xteam.xbio.top/CellMarker/). Bioinformatic analysis was performed using the OmicStudio tools at https://www.omicstudio.cn/tool.

### 4.5 Histology

Mice were euthanized and perfused intracardially with saline; brains were removed and post‐fixed for 24 h in 4% paraformaldehyde; then dehydration was carried out using sequential alcohol washes with 80%, 95%, 100%, and 100% ethanol, respectively. Xylene was used to perforate the brain tissue. The brains were molded using paraffin wax, and 5 μm tissue sections were prepared and collected on poly‐L‐lysine‐coated slides. Hematoxylin and eosin (HE) staining was performed after dewaxing and rehydration. Slides were checked under a light microscope, and images were captured with Caseviewer software (version 2.4, 3DHISTECH Ltd.).

### 4.6 BBB integrity assay

An vivo BBB permeability assay by Evans Blue (EB) was previously published by us.^23^ Mice were injected intravenous (i.v.) with 100 μL of 1% EB in phosphate‐buffered saline (PBS). Eight hours later, mice were lethally anesthetized and perfused intracardially with saline to remove intravascular EB. Brains were removed, and blue in the brain parenchyma indicates severe BBB opening and breakdown.

### 4.7 Immunofluorescence staining

To perform immunofluorescence staining, brain sections were treated with dewaxing and tissue antigen recovery, then permeabilized with 0.2% Triton X‐100 and blocked in 10% normal goat serum for 1 h at 37°C. After blocking, brain sections were incubated with primary antibodies recognizing CD3, CD8a, NeuN, IBA‐1, CCR2, Ki‐67, CCL‐2, PD‐1, PD‐L1, and STAT‐1 in 5% nonfat dry milk for overnight at 4°C. Sections were washed with PBS with 0.2% Tween‐20 (PBST) 3 times for 5 min at 20°C. Corresponding secondary antibodies where goat anti‐rat‐PE (Southern Biotech, Cat.3050‐09), Goat anti‐rabbit‐PE (Abcam, Cat.ab72465), goat anti‐rabbit‐dylight 488 (Rockland, Cat.611‐141‐002), and goat anti‐rat Alexa Fluor 488 (Abcam, Cat.ab150165) in PBST for 1 h at 37°C. After washing with PBST, the nuclei were stained with DAPI (Abcam, Cat.ab104139) for 15 min at 20°C, and a mounting medium (Life Technologies) was employed to cover the sections. Slides were examined by a fluorescence microscope (Olympus BX51), and images were captured with Caseviewer software. The mean fluorescence intensity was calculated by ImageJ software (version 1.53).

### 4.8 TdT‐mediated dUTP‐biotin nick end labeling (TUNEL) assay

Brain parenchyma cell damage and apoptosis were achieved by detecting DNA strand breaks. Those DNA breaks can be identified by labeling the free 3′‐OH terminal with modified nucleotides in an enzymatic reaction. Terminal deoxynucleotidyl transferase (TdT) can catalyze DIG‐dUTP to free 3’‐OH DNA ends. The TUNEL assay of the brain section was used by the In Situ Cell Apoptosis Detection Kit (Beyotime, Cat.C1088) following the instructions.

### 4.9 Flow cytometry

Purified brain‐infiltrated T cells, splenic CD8^+^ T cells, or digested primary astrocytes were resuspended in 2% fetal bovine serum (FBS)/PBS buffer and then filtered through 40 ‐μm cell strainers. For surface antigens, such as CD3, CD8, CD69, PD‐1, CD80, CD86, MHC I, and MHC II, single cells were stained with corresponding antibodies and incubated in the dark on ice for 20 min. Cells were washed twice and resuspended in 2% FBS or PBS, then detected by flow cytometry. For intracellular cytokines staining, such as IFN‐γ, cell surface antigen staining was performed first, then fixation (Biolegend, Cat.420801) and permeabilization (Biolegend, Cat.421002) were performed under recommended usage. Fixed and permeabilized cells stained with PE‐labeled anti‐mouse IFN‐γ antibody, detected by flow cytometry. For nucleus antigen Ki‐67 staining, cell surface antigen staining was performed first; fixation and permeabilization were used with 70% ethanol, then stained with PE‐labeled anti‐mouse ki‐67 antibody. Cell samples were analyzed on an EXFLOW flow cytometer device (DAKEWE, China). Data analysis was performed using FlowJo software (version 7.6.1).

### 4.10 Purification of brain‐infiltrated T cells

The technique for isolating brain‐infiltrated T cells was published before.^23^ Mice were euthanized and perfused intracardially with saline to remove nonadhered RBCs and leukocytes from the brain. Brains were removed and homogenized by passing through a 23‐gauge needle; the homogenates were centrifuged at 800 g for 10 min at 4°C, and pellets were dissolved in RPMI medium containing 0.5 mg/mL collagenase II and 10 μg/mL DNase I for 30 min at 20°C. The tissue extract was then centrifuged at 800 g for 5 min. The pelleted cells were further purified on a 30% Percoll gradient (GE Healthcare, Cat.17‐0891). The upper Percoll layers containing myelin debris and cells other than leukocytes were carefully removed, and the cell pellet containing brain‐infiltrated leukocytes (BIL) was resuspended in PBS and counted. T cells were negatively isolated from BILs with immunomagnetic beads according to the manufacturer's instructions (BD, Cat.557793), and the proportion of CD8^+^ T cells was determined by flow cytometry.

### 4.11 Enzyme‐linked immunosorbent assay (ELISA)

In the ex vivo killing assay, the supernatant was collected, and granzyme B secretion by CD8^+^ T cells was analyzed by the mouse Granzyme B ELISA Kit (Genie, Cat.MOFI00260) according to the manufacturer's protocol. The absorbances at 450 nm and 630 nm were immediately measured by an automatic microplate reader (Bio‐Rad, Model 680) after adding the stop solution.

### 4.12 Western blotting

The total protein of the brainstem or primary cells was extracted by RIPA with PMSF (Beyotime, Cat.ST506) and determined via the BCA Protein Assay Kit (Beyotime, Cat.P0012), then mixed with 5 × protein loading buffer (Beyotime, Cat. P0280) and boiled at 98°C for 15 min. Equal amounts of protein were separated on SDS‐PAGE gels and then transferred to the PVDF membrane (Millipore). After blocking with 5% nonfat dry milk, the PVDF membrane was incubated with primary antibodies against mouse PD‐L1, p‐STAT‐1, and β‐actin (Proteintech, Cat.66009) at 4°C overnight and then hybridized with the corresponding HRP‐labeled secondary antibody at 37°C for 1 h. The intensity of the bands was analyzed with a chemiluminescence kit (Millipore).

### 4.13 Astrocytes primary culture

The rains of mice aged days 1–3 were extracted and washed with ice‐cold Hanks' balanced salt solution (HBSS). Then tissues were transferred to ice‐cold DMEM supplemental, and meninges were removed. Cleaned cortices were minced into small pieces with forceps and then dissociated mechanically by polished Pasteur pipettes until tissue pieces disappeared. Cell debris and aggregates were removed by passing the single‐cell suspension through a 40 ‐μm cell strainer. After centrifugation at 800 g for 5 min, the supernatant was discarded. The cell mass was resuspended in 2 mL DMEM with 10% FBS and seeded on poly‐L‐lysine‐coated T75 flasks (about 7 brains/T75 flask). After 10–14 days, cells proliferated to form confluent, microglia were shaken off at 220 rpm/min overnight and collected, and astrocytes were passaged to T75 flasks. For cytokine activation of astrocytes, 20 ng/mL IFN‐γ was added to the cell medium 24 h before subsequent analysis.

### 4.14 Astrocytes and CD8^+^ T cell cocultured model

Primary astrocytes were isolated and purified as described above, then activated with 20 ng/mL IFN‐γ and pRBCs for 24 h to mimic the pro‐inflammatory microenvironment typically observed in the brain of ECM mice. For the next step, IFN‐γ and pRBCs were removed by washing with PBS. Brain‐infiltrated CD8^+^ T cells were isolated (BD, Cat. 558,471) from ECM mice (7 dpi) and cocultured with stimulated astrocytes (E: T = 10:1) for another 24 h. Astrocytes and CD8^+^ T cells were collected, respectively. Activation of astrocytes was evaluated by expression of CD80, CD86, MHC I, and MHC II with flow cytometry, and activation of CD8^+^ T cells was evaluated by early activation marker CD69 expression with flow cytometry. LDH and granzyme B content in culture supernatants were measured to detect the cytotoxicity of brain‐infiltrated CD8^+^ T cells (Figure 6B).

### 4.15 Statistical analyses

The results were reported as the means ± standard error of the mean (SEM) of at least three independent experiments. Data were analyzed and graphs generated by GraphPad Prism (version 8.02, GraphPad Software). Kaplan‐Meier survival curves were generated and analyzed by the log‐rank Mantel‐Cox tests for the mouse survival study. Student's *t*‐test and one‐way ANOVA were used to determine the statistical significance for comparisons of 2 or more groups; *p* < 0.05 was considered statistically significant, *p < 0.05, **p < 0.01, ***p<0.001.

## AUTHOR CONTRIBUTIONS

YZ and YL conceived and designed the experiments, JW, QZ, YS, and JL performed the experiments; YW, NZ, XW, and KY conducted the in vitro assays; JW, QZ, and YS analyzed the data; GT and QZ contributed reagents and materials; JW and QZ wrote the manuscript. All authors read and approved the paper.

## FUNDING INFORMATION

This work was supported by the National Natural Science Foundation of China (NO. 82002158, 82072298, 81702019) and the Natural Science Foundation of Shaanxi Province (NO. 2019JQ‐408, 2017SF‐192).

## CONFLICT OF INTEREST STATEMENT

The authors declare no competing interests.

## Supporting information


Figure S1.

Figure S2.

Figure S3.

Figure S4.

Figure S5.

Figure S6.

## Data Availability

The dataset generated during the current study is available from the corresponding author upon reasonable request. All raw sequencing reads for scRNA‐seq data can be found under BioProject PRJNA735877 (http://www.ncbi.nlm.nih.gov/bioproject).
