# Supplementary figures and images for: CD8+ T cell infiltration and proliferation in the brainstem during experimental cerebral malaria
Source: CNS Neurosci Ther. 2023 Sep 12;30(3):e14431. doi: 10.1111/cns.14431 (PMC10916431; doi:10.1111/cns.14431)

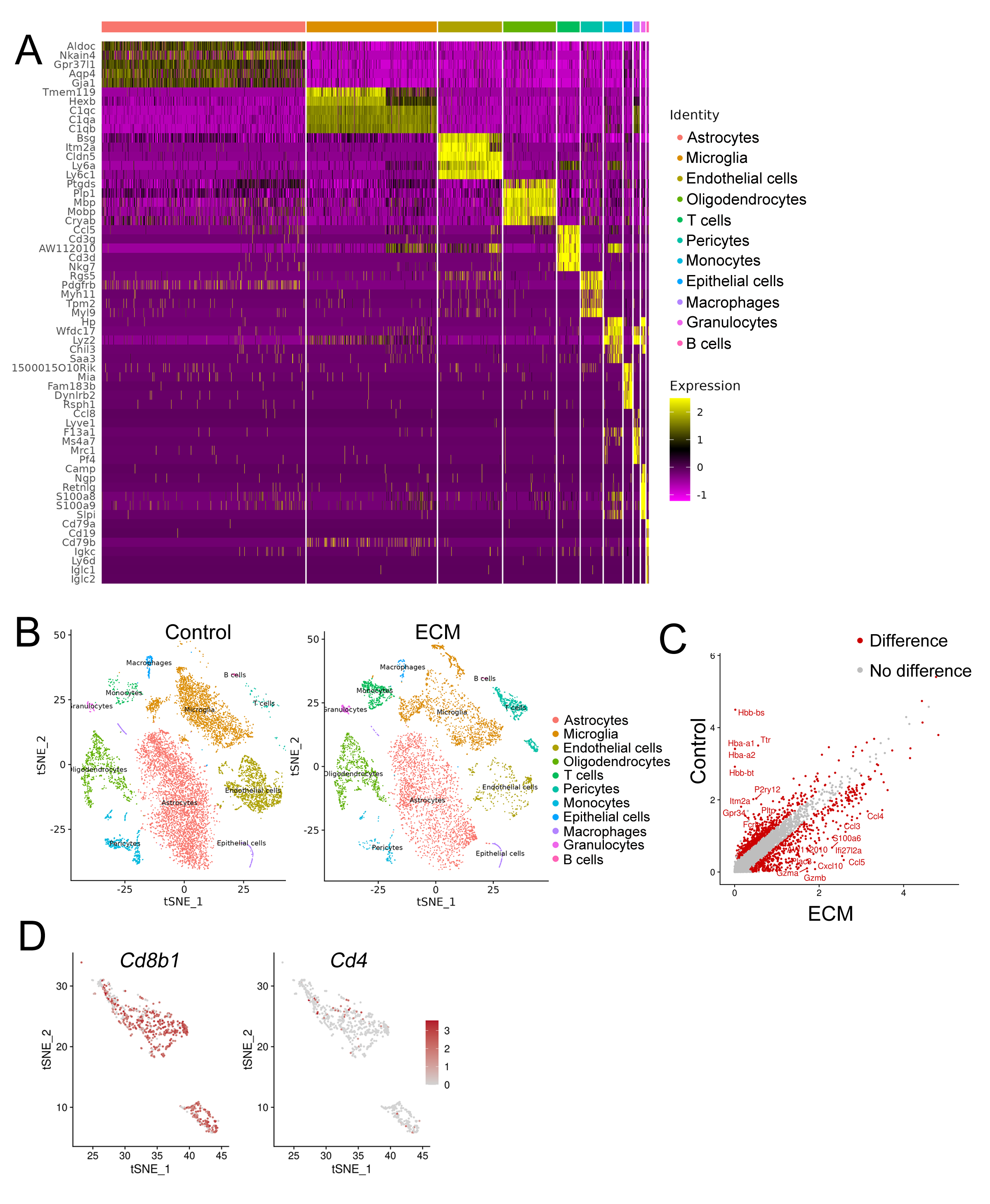

Supplement: Supplementary file 1 — Figure S1. [file CNS-30-e14431-s004.tif]

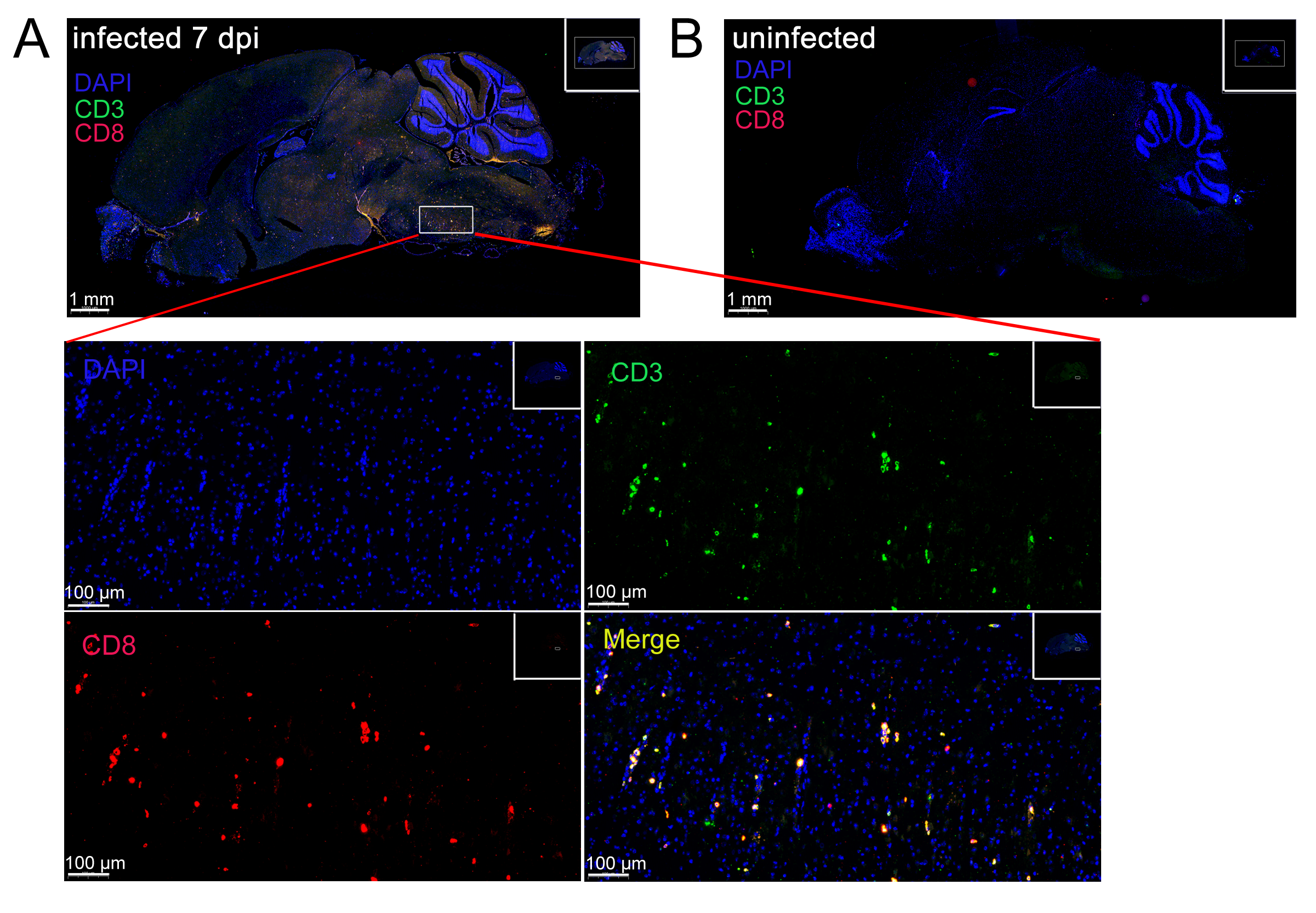

Supplement: Supplementary file 2 — Figure S2. [file CNS-30-e14431-s005.tif]

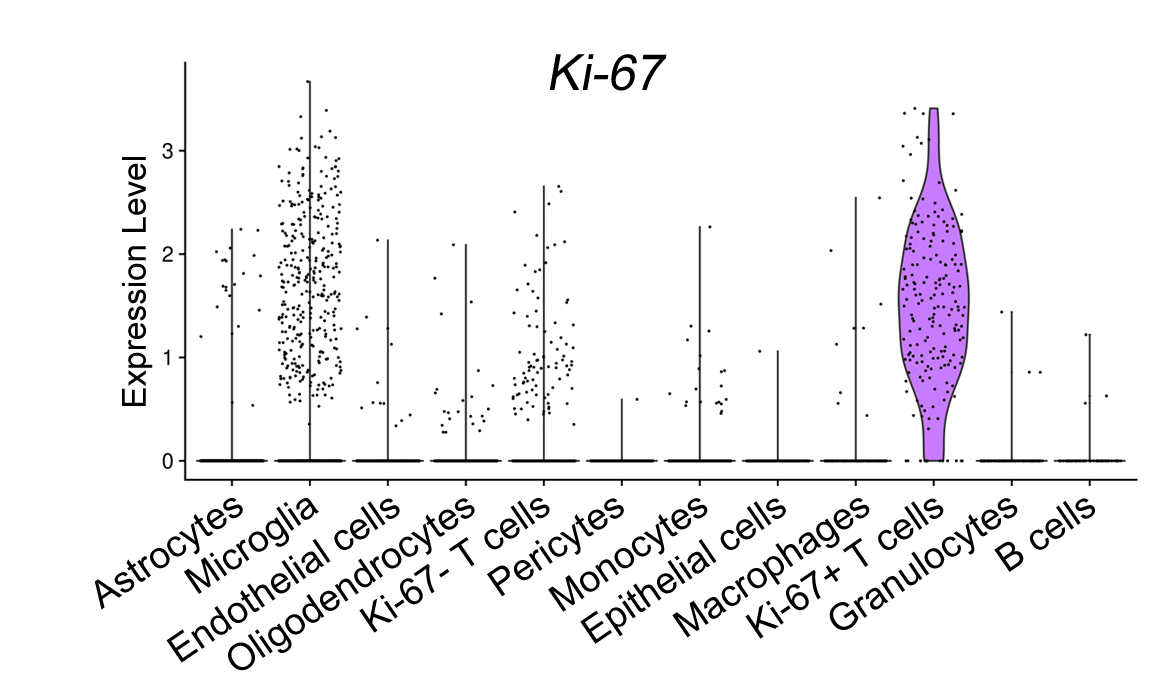

Supplement: Supplementary file 3 — Figure S3. [file CNS-30-e14431-s002.tif]

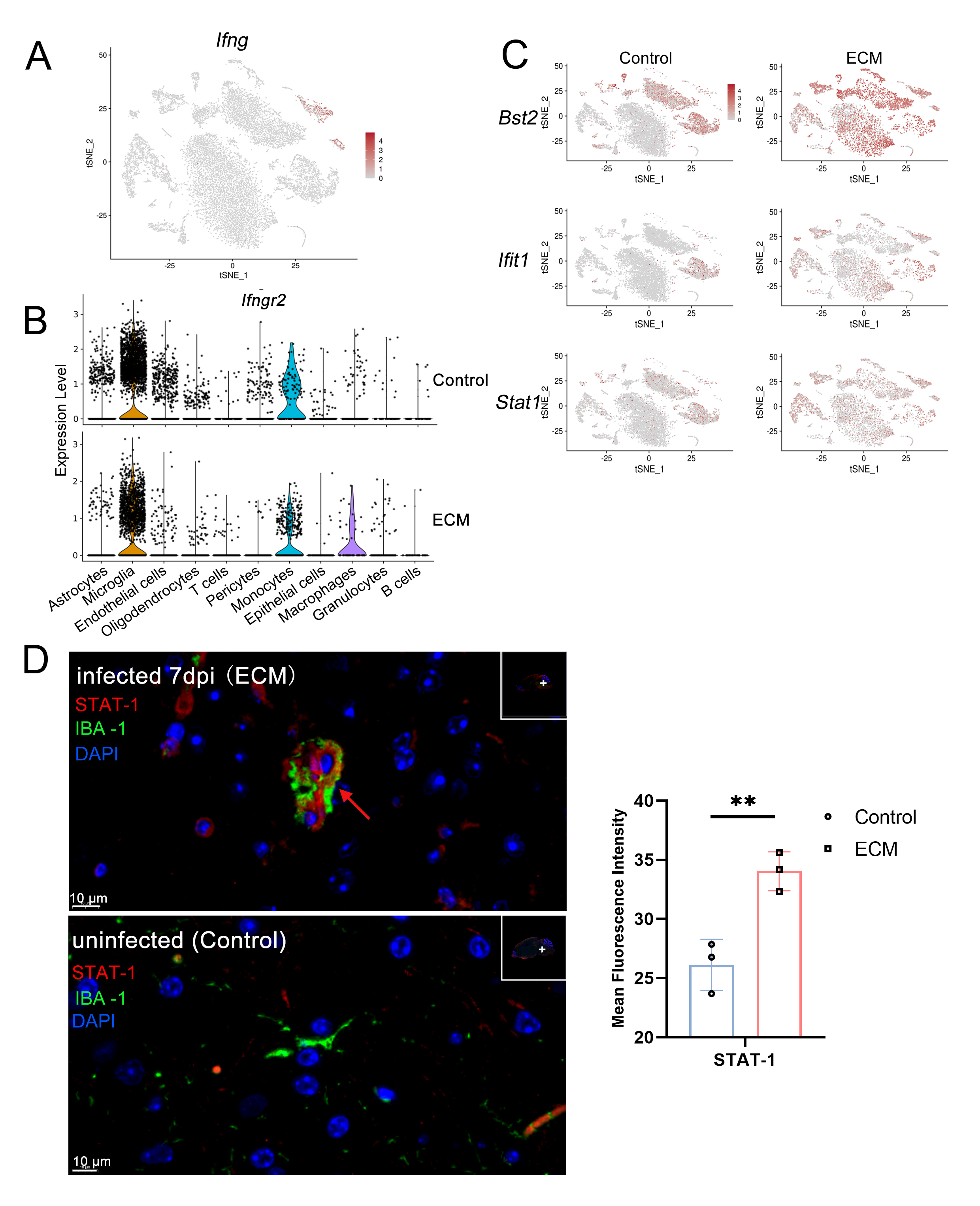

Supplement: Supplementary file 4 — Figure S4. [file CNS-30-e14431-s003.tif]

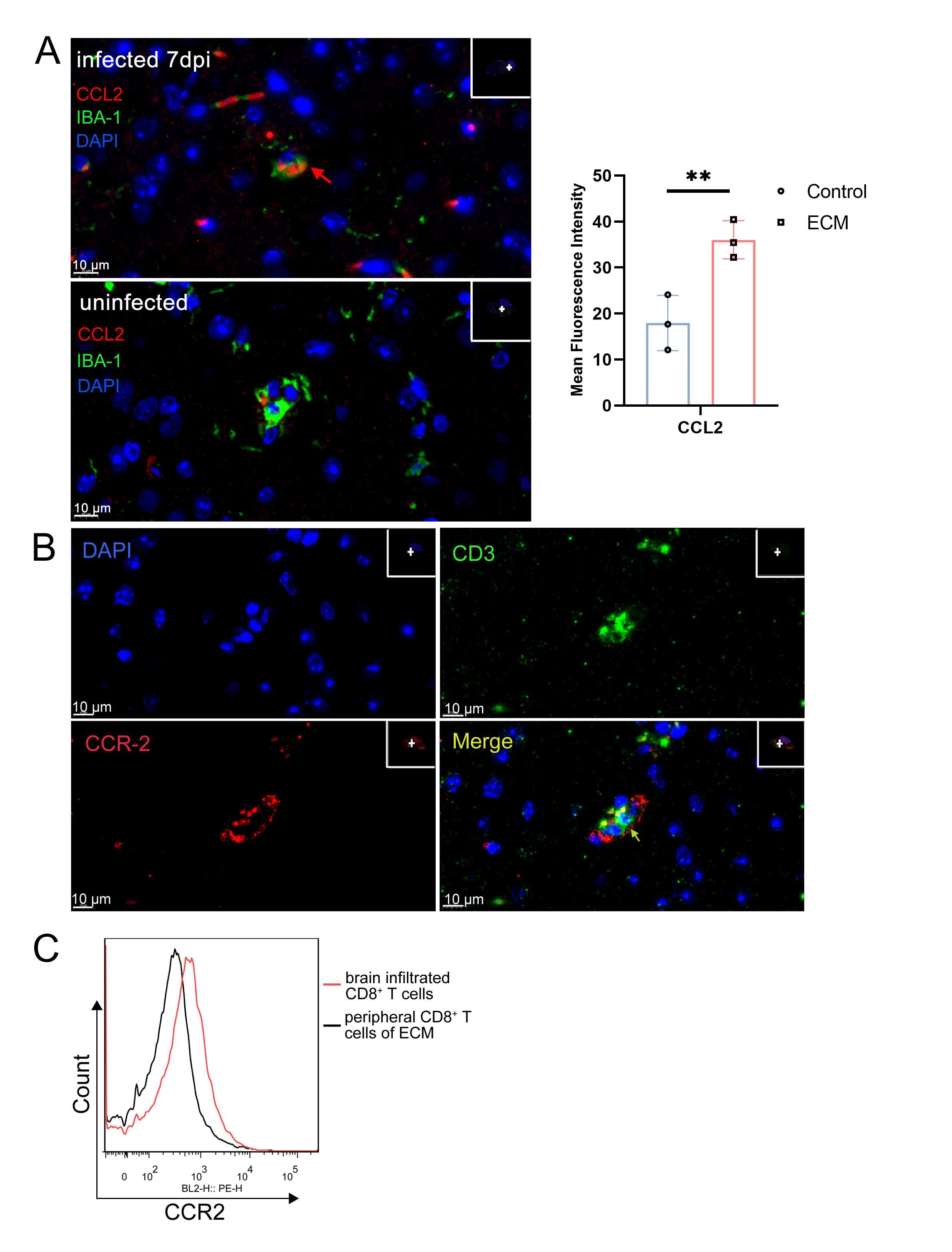

Supplement: Supplementary file 5 — Figure S5. [file CNS-30-e14431-s001.tif]

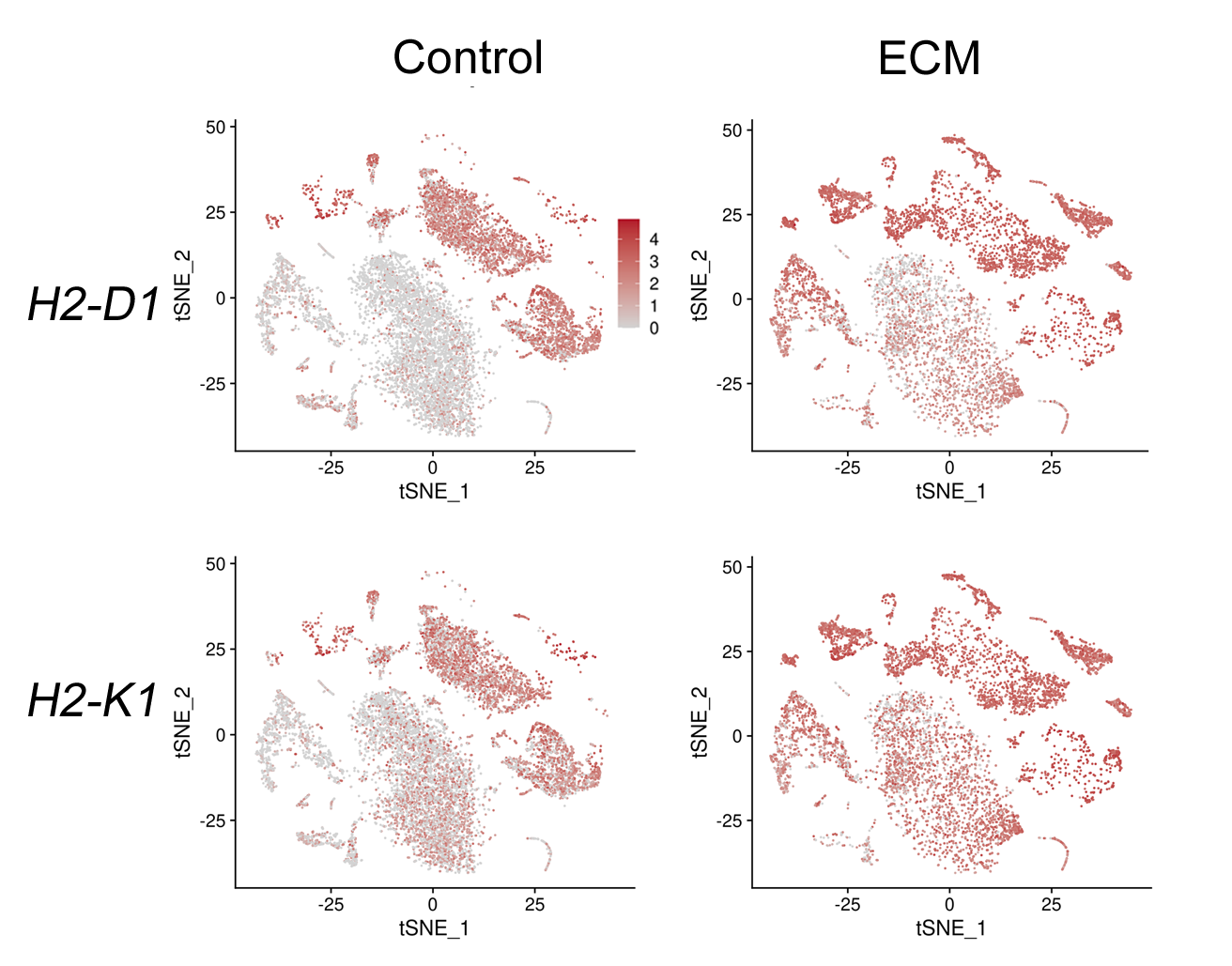

Supplement: Supplementary file 6 — Figure S6. [file CNS-30-e14431-s006.tif]
